# HLA Class I Expression and Its Alteration by Preoperative Hyperthermo-Chemoradiotherapy in Patients with Rectal Cancer

**DOI:** 10.1371/journal.pone.0108122

**Published:** 2014-09-26

**Authors:** Hiro Sato, Yoshiyuki Suzuki, Munenori Ide, Toshihide Katoh, Shin-ei Noda, Ken Ando, Takahiro Oike, Yuya Yoshimoto, Noriyuki Okonogi, Kousaku Mimura, Takayuki Asao, Hiroyuki Kuwano, Takashi Nakano

**Affiliations:** 1 Department of Radiation Oncology, Gunma University Graduate School of Medicine, Maebashi, Japan; 2 Department of Human Pathology, Gunma University Graduate School of Medicine, Maebashi, Japan; 3 Department of General Surgical Science, Gunma University Graduate School of Medicine, Maebashi, Japan; 4 Department of Surgery, National University of Singapore, Singapore, Singapore; 5 Department of Oncology Clinical Development, Gunma University Graduate School of Medicine, Maebashi, Japan; Karolinska Institutet, Sweden

## Abstract

**Objective:**

Enhancing immunologic responses, including human leukocyte antigen (HLA) class I expression on tumor cells and recognition and elimination of tumor cells by tumor-specific cytotoxic T lymphocyte (CTL), is considered a novel concept of radiotherapy. The present study examined patients who underwent preoperative hyperthermo-chemoradiotherapy (HCRT) for locally advanced rectal cancer to assess the correlation between HLA class I expression and clinical outcome.

**Materials and Methods:**

Seventy-eight patients with locally advanced rectal adenocarcinoma who received preoperative HCRT were enrolled. The median age of the patients was 64 years (range, 33–85 years) and 4, 18, and 56 patients had clinical stage I, II and III disease, respectively. Formalin-fixed and paraffin-embedded tissues excised before and after HCRT were subjected to immunohistochemical analysis with an anti-HLA class I-A, B, C antibody. HLA class I expression was graded according to tumor cell positivity.

**Results:**

In pre-HCRT, the number of specimens categorized as Grade 0 and 1 were 19 (24%) and 58 (74%), respectively. Only 1 patient (1%) showed Grade 2 expression. However, 6 (8%), 27 (35%), 7 (9%), and 12 (15%) post-HCRT specimens were graded as Grade 0, 1, 2, and 3, respectively. There was a significant increase in HLA class I expression in post-HCRT specimens (p<0.01). However, neither pre- nor post-HCRT HLA class I expression affected overall survival and distant metastasis-free survival in clinical stage III patients. Univariate analysis revealed that Post-HCRT HLA class I expression showed a significant negative relationship with LC (p<0.05). Nevertheless, multivariate analysis showed that there was no correlation between HLA class I expression and clinical outcome.

**Conclusion:**

HCRT increased HLA class I expression in rectal cancer patients. However, multivariate analysis failed to show any correlation between the level of HLA class I expression and prognosis.

## Introduction

Accumulating evidence supports the importance of cell-mediated immunity for controlling tumor growth and eliminating distant metastases [1]. Human leukocyte antigen (HLA) class I molecules and tumor antigen-specific cytotoxic T lymphocytes (CTL) play crucial roles in these responses. HLA class I molecules expressed by tumor cells present tumor-derived antigens to CTL, which then kill the target cells [2]. Many studies report a significant correlation between clinical prognosis and the expression of HLA class I by esophageal, non-small cell lung, head and neck squamous cell, and bladder cancers [3]–[8]. Moreover, the majority of these reports conclude that patients with low HLA class I-expressing cancers have a poorer prognosis than those with high-expressing cancers.

Radiotherapy is a major form of anti-cancer therapy, and is used to treat many types of cancer, regardless of clinical stage. It is generally accepted that irradiation induces cell death via apoptosis and/or necrosis by damaging (either directly or indirectly) DNA. However, recent studies suggest that radiation-induced immunogenic cell death is a concept different from the more “traditional” radiation-induced cell death [9]–[11]. The concept underlying radiation-induced immunogenic cell death is based on the induction of anti-tumor immune CTL responses by radiotherapy with modification of the immunogenic epitopes on tumor HLA class I molecules. Radiotherapy increases the expression of HLA class I molecules by human melanoma cells and by kidney and subcutaneous cells in HLA-A2 transgenic mouse [12]. By contrast, Speetjens et al. reported that irradiation did not effect HLA class I expression by rectal cancer cells [13]. However, few reports have examined the correlation between radiotherapy and HLA expression on tumor cells in a clinical setting [12], [14], [15]. Moreover, to the best of our knowledge, no studies have examined changes in HLA class I expression by tumor cells in the same patient before and after radiotherapy.

We previously treated rectal cancer patients with preoperative hyperthermo-chemoradiotherapy (HCRT) for several years and reported the clinical outcomes [16], [17]. Here, we obtained samples from the same patients to examine (i) HLA class I expression on rectal cancer cells, (ii) changes in HLA class I expression induced by HCRT, and (iii) the relationship between HLA class I expression and prognosis.

## Materials and Methods

### Ethics statement

All patients provided their written informed consent to participate in this study. This study was approved by the institutional review board of Gunma University Graduate School of Medicine, Gunma, Japan (IRB approval number is 1051). Anonymity of the patients was preserved.

### Patients and specimens

Seventy-eight patients with locally advanced rectal adenocarcinoma, who were consecutively treated with HCRT followed by surgery at Gunma University Hospital between 2003 and 2011, were enrolled in the study. Pre- and post-HCRT specimens were available for all patients.

The characteristics of the patient’s and the tumors are summarized in Table 1. The median age of the patients at the start of HCRT was 64 years (range, 33–85 years). The mean and median follow up periods were 45 and 40 months (range, 3–103 months), respectively. For diagnostic workup, all patients underwent computed tomography (CT) of the abdomen and thorax to facilitate staging of regional and distant metastases. T stage was determined by CT and magnetic resonance imaging (MRI), particularly T2 weighted images, according to the TNM Classification of Malignant Tumors (UICC, 7th edition). Overall, 4, 18 and 56 patients were classified with stage I, II and III disease, respectively. Samples from the 56 patients with stage III disease were further examined to determine the correlation between HLA class I expression and prognosis.

**Table 1:** Patient and tumor characteristics.

Patient characteristics	
**Age (years)**	
Median (range)	64 (33–85)
**Gender**	
Male	55 (71%)
Female	23 (29%)
**Tumor stage**	
T1	0 (0%)
T2	9 (12%)
T3	47 (60%)
T4a	12 (15%)
T4b	10 (13%)
**Lymph-node metastasis**	
N0	21 (27%)
N1	28 (36%)
N2	29 (37%)
**Clinical stage**	
1	4 (5%)
2	18 (23%)
3	56 (72%)

Tissue specimens were excised endoscopically from rectal tumors before HCRT and then surgically after HCRT (median, 10 weeks; mean, 12 weeks; range, 1–54 weeks). Excised tissues were then fixed in 10% formalin for 24 h and embedded in paraffin. HLA class I expression was examined by immunohistochemistry.

### Treatments

All 78 patients underwent preoperative HCRT, as previously reported [16]. Briefly, external beam radiotherapy (X rays; 10 MV) was delivered using an anteroposterior or three- or four-field box technique. The clinical target volume (CTV) comprised the primary tumor and the entire mesorectal tissue. The total radiation dose was 40–54 Gy (median, 50 Gy), with daily fractions of 2 Gy. Seventy-four patients also received 5-fluorouracil (5-FU) and Levofolinate, three received UFT and leucovorin, and one received Capecitabine.

Hyperthermia treatment was performed on a weekly basis (2–6 sessions (median, 5 sessions) each lasting 30 min) using capacitive heating equipment and a radiofrequency of 8 MHz (Thermotron-RF 8, Yamamoto Vinita Co. Ltd., Japan).

Surgery (mainly total mesorectal excision) was performed at 1–54 weeks (median, 10 weeks) after the completion of HCRT. Seventy-five patients (96%) underwent surgery within 17 weeks. Several patients who showed a clinical complete response to HCRT refused surgery; therefore, surgery was performed in only 3 patients (4%) of patients after more than 34 weeks.

### Immunostaining for HLA class I

Formalin-fixed and paraffin-embedded specimens were used for immunohistochemical analysis. Tissue sections were deparaffinized and subjected to antigen retrieval with epitope retrieval solution (Target Retrieval Solution, Citrate pH 6; Dako, Glostrup, Denmark) at 121°C for 20 min. Endogenous peroxidase activity was blocked by Dako REAL (Dako). A primary antibody against the HLA class I-A, B, C heavy chain, EMR8-5 (Hokudo, Sapporo, Japan), which does not recognize the HLA class I heavy chain-β2-microglobulin-peptide complex, was diluted (1∶70) in Antibody Diluent Solution with Background Reducing Components (Dako) and incubated with the tissue sections at 4°C overnight [18]. The sections were then incubated with a Histofine Simple Stain MAX-PO kit (Nichirei, Tokyo, Japan) for 30 min. Finally, the sections were treated with 3, 30-diaminobenzidine (DAB, Dako) for 1 min, and counterstained with hematoxylin. Specimens of normal lymph node served as positive controls. The isotype matched irrelevant immunoglobulin (Negative Control Mouse IgG1; Dako) was used as the negative control.

Two authors (HS and MI, both blinded to the clinical and pathological data) assessed HLA class I positivity in a semi-quantitative manner. Staining was classified as follows: Grade 0, <10% stained cells; Grade 2, 10–50% stained cells; Grade 3, 50–90% stained cells; Grade 4, >90% stained cells. The relationship between HLA class I expression and clinical prognosis was assessed by dividing the post-HCRT specimens into two groups, as previously described [6], [13], [19]: Grades 0–1 and Grades 2–3. The relationship between HLA class I expression and clinical prognosis was not examined in pre-HCRT specimens because only one patient showed Grade 2–3 expression prior to HCRT.

### Statistical analysis

Survival was measured in days, starting from Day 1 of HCRT to the time of death or final follow up. Univariate analysis of the actuarial overall survival rate (OS), the local control rate (LC), and the distant metastasis-free survival rate (DMFS) for each clinicopathological factor were analyzed using the Kaplan-Meier method, and differences between survival curves were analyzed using the log-rank test. Multivariate analyses using Cox’s proportional hazards model were performed to examine the correlation between prognosis and multiple clinicopathological variables. Differences in HLA class I expression between pre- and post-HCRT specimens were examined using the Wilcoxon signed rank test. Differences were considered significant at P<0.05. All statistical analyses were performed using SPSS software, version 21 (SPSS Japan Inc., Tokyo, Japan).

## Results

### HLA class I expression

**Figure 1:**
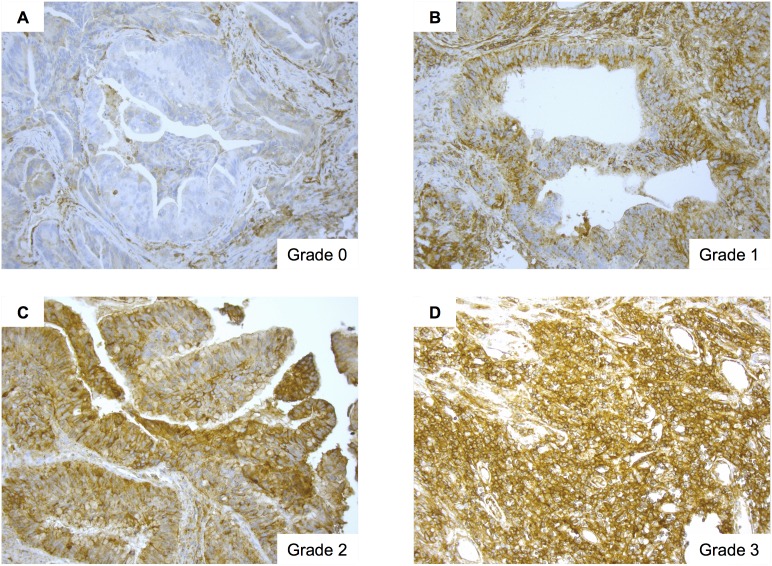
Human leukocyte antigen (HLA) class I expression in tumor cells. Representative images showing HLA class I expression by rectal cancer cells. A, Grade 0 expression. B, Grade 1 expression. C, Grade 2 expression. D, Grade 3 expression. Magnification, ×200.

Figure 1 shows HLA class I expression by rectal tumor cells. The number of pre-HCRT specimens categorized as Grade 0, 1, 2, and 3 was 19 (24%), 58 (74%), 1 (1%), and 0 (0%), respectively. The number of post-HCRT specimens categorized as Grade 0, 1, 2, and 3 was 6 (8%), 27 (35%), 7 (9%), and 12 (15%), respectively. Twenty-six patients (33%) showed a pathological complete response (pCR) post-HCRT. Table 2 shows that HLA class I expression correlated with patient age (<60 *vs.* ≤60), but not with other any other tumor characteristics or disease stage.

**Table 2:** Relationship between pre-HCRT HLA class I expression and patient/tumor characteristics.

		Grade of HLA class I expression				
	Patient no. (%)	0	1	2	3	P-value
		No. (%)	No. (%)	No. (%)	No. (%)	
**All**	78	19 (24)	58 (74)	1 (1)	0 (0)	
**Gender**						
Male	55 (71)	15 (27)	40 (72)	0 (0)	0 (0)	0.68
Female	23 (29)	4 (17)	9 (78)	1 (4)	0 (0)	
**Age**						
<60	27 (35)	2 (7)	24 (89)	1 (4)	0 (0)	<0.05
≥60	51 (65)	17 (33)	34 (67)	0 (0)	0 (0)	
**T stage**						
1	0 (0)	0 (0)	0 (0)	0 (0)	0 (0)	0.17
2	9 (12)	0 (0)	9 (100)	0 (0)	0 (0)	
3	47 (60)	16 (34)	30 (64)	1 (2)	0 (0)	
4a	12 (15)	2 (17)	10 (83)	0 (0)	0 (0)	
4b	10 (13)	1 (10)	9 (90)	0 (0)	0 (0)	
**N stage**						
0	21 (27)	6 (29)	14 (67)	1 (5)	0 (0)	0.60
1	28 (36)	7 (25)	21 (75)	0 (0)	0 (0)	
2	29 (37)	6 (21)	23 (79)	0 (0)	0 (0)	
**TNM stage**						
I	4 (5)	0 (0)	4 (100)	0 (0)	0 (0)	0.21
II	18 (23)	6 (33)	11 (61)	1 (6)	0 (0)	
III	56 (72)	13 (23)	43 (77)	0 (0)	0 (0)	

HCRT, hyperthermo-chemoradiotherapy; HLA, human leukocyte antigen.

### Changes in HLA class I expression induced by HCRT

**Figure 2:**
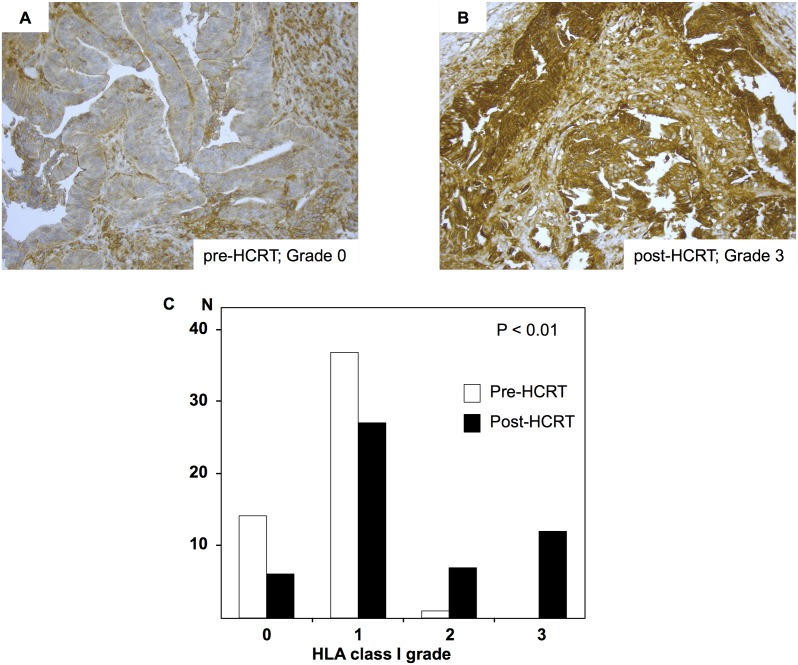
Changes in Human leukocyte antigen (HLA) class I expression after preoperative hyperthermo-chemoradiotherapy (HCRT). Grades of HLA class I expression in samples from the same patient. A, pre-HCRT specimen; Grade 0. B, post-HCRT specimen; Grade 3. Magnification, ×200. C, HLA class I expression in pre- and post-HCRT specimens. Predictive value was tested using the Wilcoxon signed rank test.

Overall, 26 patients achieved pCR after HCRT. Changes in HLA class I expression were evaluated in tumor samples from the remaining 52 patients. Of these, 26 (50%) showed increased HLA class I expression, 3 (6%) showed reduced expression and 23 (44%) showed no change. The increase in HLA class I expression after HCRT was significant (p<0.01) (Figure 2). Of the 26 patients that achieved pCR, 5 (19%) showed Grade 0 expression pre-HCRT and 21 (81%) showed Grade 1 expression. There was no significant correlation between the Grade of HLA class I expression pre-HCRT and pCR.

### Correlation between HLA class I expression and prognosis

The 5 year OS, LC, and DMFS for all 78 patients with Grade 0–1 post-HCRT HLA class I expression were 76%, 94%, and 76%, respectively, whereas the 5 year OS, LC, and DMFS of patients with Grade 2–3 post-HCRT HLA class I expression were 79%, 74%, and 79%, respectively (Table 3). There was no significant correlation between the three outcome parameters and the Grade of HLA class I expression. The pCR induced by HCRT showed a significant relationship with good LC (p<0.05).

**Table 3:** Univariate analysis of 5 year survival rates after hyperthermo-chemoradiotherapy (HRCT).

Factors	N (%)	OS		LC		DMFS	
		Rate (%)	P-value	Rate (%)	P-value	Rate (%)	P-value
**Age**							
<60	27 (35)	85	0.32	93	0.65	78	0.95
≥60	51 (65)	77		90		80	
**Gender**							
Male	55 (71)	78	0.66	87	0.08	82	0.49
Female	23 (30)	83		100		74	
**Clinical stage**							
1	4 (5)	100	0.55	75	0.19	100	0.63
2	18 (23)	72		83		78	
3	56 (80)	80		95		79	
**HLA (Pre-HCRT)**							
Grade 0, 1	77 (99)	79	0.56	91	0.73	81	0.06
Grade 2, 3	1 (1)	100		100		0	
**HLA (Post-HCRT)**							
Grade 0, 1	33 (64)	76	0.60	94	0.86	76	0.63
Grade 2, 3	19 (37)	79		74		79	
**Pathological effect**							
pCR	26 (33)	85	0.42	100	<0.05	85	0.55
No pCR	52 (67)	77		87		77	

OS, overall survival; LC, local control; DMFS, distant metastasis-free survival; HLA, human leukocyte antigen.

**Figure 3:**
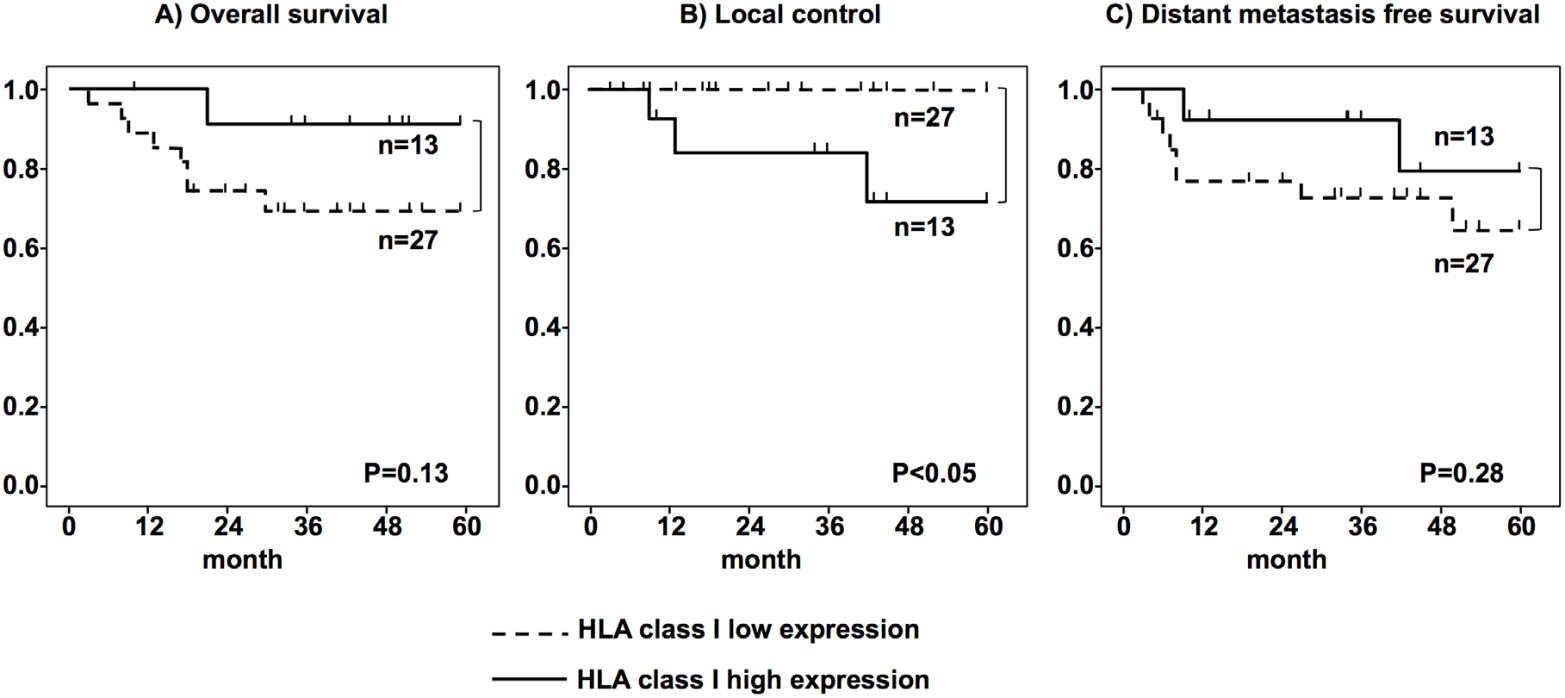
Relationship between Human leukocyte antigen (HLA) class I expression and clinical outcome. The clinical outcomes of 56 patients with stage III disease. (A; overall survival, B; local control, and C; distant metastasis-free survival). Low HLA class I expression: Grades 0 and 1; high expression: Grades 2 and 3.

Next, we further examined samples from the 56 patients with stage III disease to determine the correlation between HLA class I expression and prognosis. The 5-year OS, LC, and DMFS for stage III patients with Grade 0–1 post-HCRT HLA class I expression were 70%, 100%, and 70%, respectively, and the 5-year OS, LC, and DMFS for patients with Grade 2–3 post-HCRT HLA class I expression were 92%, 77%, and 85%, respectively (Figure 3). Post-HCRT HLA class I expression showed a significant negative relationship with LC (p<0.05). However, multivariate analysis showed that none of the variables correlated with clinical prognosis (Table 4).

**Table 4:** Multivariate analysis of 5 year survival rates after hyperthermo-chemoradiotherapy (HCRT).

Factors	OS		LC		DMFS	
	HR (95% CI)	P-value	HR (95% CI)	P-value	HR (95% CI)	P-value
Age (<60 or≥60)	0.82 (0.20–3.30)	0.79	1.18 (0.10–13.49)	0.89	0.93 (0.25–3.45)	0.92
Gender	0.86 (0.39–1.91)	0.72	0.00 (0.0–4.73E+135)	0.97	1.21 (0.61–2.41)	0.58
HLA class I (post-HCRT)	4.62 (0.57–37.47)	0.15	0.00 (0.0–7.68E+232)	0.96	2.26 (0.46–11.03)	0.31

OS, overall survival; LC, local control; DMFS, distant metastasis-free survival; CI, confidence interval; HLA, human leukocyte antigen; HR, Hazard ratio.

We next investigated examined whether the increase in HLA class I expression induced by HCRT improved the clinical outcome for stage III patients. The 5-year OS and DMFS for the “increased” group and the “constant or decreased” group were 77% and 78% (p = 0.97) and 71% and 78% (p = 0.66), respectively. There was no significant correlation between changes in HLA class I expression and OS and DMFS. On the other hand, the 5-year LC for the “increased” group and the “constant or decreased” group was 82% and 100%, respectively (p = 0.04). The LC of the “increased” HLA class I expression group was significantly worse than that of “constant or decreased” expression group.

## Discussion

The main findings of the present study were as follows: (i) 19 (24%) and 58 (74%) of tumor tissue samples from patients with rectal adenocarcinoma showed Grade 0 (<10%) or Grade 1 (10–50%) expression of HLA class I, respectively; (ii) preoperative HCRT led to a significant increase in HLA class I expression; and (iii) multivariate analysis showed no significant positive association between HLA class I expression and survival.

Speetjens et al. examined the expression of HLA class I molecules in rectal cancer [13]. They categorized HLA class I expression as follows: absent (no expression), negative (0–50% positive cells), and positive (51–100% positive cells). They reported that 9.7%, 32%, and 58% of specimens were categorized as absent, negative, or positive, respectively when stained for the HLA class I antigen by using anti-HLA antibody, HCA2, but that 2.1%, 21%, and 77% were absent, negative, or positive, respectively, when stained for the HLA class I antigen by using anti-HLA antibody, HC10. Only one patient in the present study showed >50% HLA class I positivity. This discrepancy may be partly explained by three factors. First, the primary monoclonal anti-HLA class I antibodies were different. They used HCA2 (which reacts with HLA-A (not HLA-A24), HLA-B73, and HLA-C molecules as well as HLA-E, HLA-F and HLA-G antigens), and HC10 (which reacts with HLA-B and HLA-C molecules and HLA-A10, -A28, -A29, -A30, -A31, -A32 and -A33 heavy chains) as the primary antibodies. However, we used EMR8-5, which reacts with HLA-A*2402, A*0101, A*1101, A*0201, A*0207, B*0702, B*0801, B*1501, B*3501, B*4001, B*4002, B*4006, B*4403, Cw*0102, Cw*0801, Cw*1202, and Cw*1502, as the primary antibody [18]. Second, semi-quantitative assessment is not the most accurate way to evaluate HLA class I expression. Finally, the differences may be explained by the “cancer immunoediting concept” proposed by Dunn, which describes the tumor alteration process consisted by three sequential phases (elimination, equilibrium and escape) to be acceptable to the immune system [20]. In this concept, the developing tumor cell had been eliminated by immune system before they become clinically apparent (elimination), however, a rare tumor cell was not killed by immune response, and consequently, these tumor cells emerge to cause clinically apparent disease (equilibrium and escape). Therefore, advanced cancer has tendency of low antigenic condition. Also, compared with Speetjens’s study, the present study involved more patients with locally advanced rectal adenocarcinoma (72% of patients had stage III disease). Thus, we may have found lower levels of HLA class I expression. According to the “cancer immunoediting concept”, HLA class I expression on tumor cells decreases with advancing stage.

Few reports have examined radiation-induced changes in HLA class I expression at different clinical phases; however, it has been demonstrated *in* *vitro* [12], [14], [15], [21]. Speetjens et al., found also no significant differences in HLA class I expression in tumor samples from patients treated with or without irradiation [13]. By contrast, we found that HLA class I expression in rectal cancer cells increased after HCRT and this discrepancy might be due to the radiation dose, the interval between radiotherapy and surgery, or to the different analysis methods employed. Speetjens et al., treated one group of patients with 25 Gy per five fractions within 10 days of surgery and then compared HLA expression in tumor cells from irradiated and non-irradiated patients. By contrast, we treated patients with 50 Gy per 25 fractions within 5 weeks of surgery, and the median interval between radiotherapy and surgery was 10 weeks. We then compared HLA class I expressions on tumor cells from pre- and post-HCRT specimens from the same patients. Reits et al. reported that irradiation led to a dose-dependent increase in the levels of intracellular peptides derived from existing proteins and increased protein synthesis via mTOR activation. This resulted in an increase in MHC class I expression. However, the optimal dose/fraction and the interval between irradiation and evaluation for the highest level of HLA class I expression in the clinical phase is unknown.

On the other hand, changes in HLA class I expression might by induced by factors other than radiation, e.g., chemotherapy and/or hyperthermia. Gamerio et al. reported that hyperthermia led to a significant increase in major histocompatibility complex (MHC) class I protein expression on the surface of murine colon adenocarcinoma cells that expressed human CEA [22]. In addition, Ohtsukasa et al. reported that anti-cancer drugs (e.g., 5-FU, SN-38, and CDDP) induced a marked increase in MHC class I expression by human colon cancer cell lines cells (COLO 201) *in* *vitro* [23]. Furthermore, they also reported that anti-cancer drugs induced MHC class I expression in murine colon tumor cells *in* *vivo*. They thought that this change might be induced by the chemotherapy-induced production of endogenous proteins and/or to increased transport of accumulated material to the cell membrane.

These results suggest that the increased HLA class I expression observed in the present study was augmented by a combination of radiotherapy, chemotherapy, and hyperthermia. Further studies are needed to understand the mechanisms underlying increases in HLA class I expression induced by different treatments; however, this is the first report to show that pre-HCRT increases HLA class I expression in a clinical setting. This result suggests that combination radiotherapy with HLA class I-mediated immunotherapy may amplify weak anti-tumor immune responses caused by loss of HLA class I expression, which is a major mechanism by which tumor cells escape immunosurveillance.

We expected that patients bearing tumors with high HLA class I expression would have a favorable clinical prognosis; however, when we performed univariate analysis, we found no significant positive association between HLA class I expression and OS or DMFS in stage III patients. Moreover, LC showed a significant negative association with post-HCRT HLA class I expression in stage III patients. In contrast to the results presented herein, most previous studies report that patients with tumors showing high HLA class I expression had a better prognosis [3]–[8]. Additionally, when we investigated the correlation between HCRT-induced changes in HLA class I expression and OS, LC, and DMFS, we found that LC showed a significant negative correlation with increased HLA class I expression. There are several reasons for these discrepancies, as follows: (i) surgical factors may have a stronger impact on clinical prognosis than pre-HCRT-induced HLA class I expression and its immunological effect; (ii) the immunological factors that affect clinical prognosis are quite complex (the effect of HLA class I expression was not strong enough to affect clinical prognosis); and (iii) the number of stage III patients (56) was insufficient to enable a meaningful evaluation of clinical prognosis. Therefore, further studies are required to establish the association between HLA class I expression and prognosis in rectal cancer patients.

In conclusion, the present study suggests that HCRT may increase HLA class I expression by rectal cancer cells; however, multivariate analysis did not show an association between HLA class I expression and prognosis.
